# Effect of hypoglycaemia on measures of myocardial blood flow and myocardial injury in adults with and without type 1 diabetes: A prospective, randomised, open‐label, blinded endpoint, cross‐over study

**DOI:** 10.1002/edm2.258

**Published:** 2021-05-07

**Authors:** Radzi M. Noh, Alex J. Graveling, Ninian N. Lang, Audrey C. White, Kuan K. Lee, Nicholas L. Mills, David E. Newby, Chim C. Lang, Brian M. Frier

**Affiliations:** ^1^ Department of Diabetes Royal Infirmary of Edinburgh Edinburgh UK; ^2^ Centre for Cardiovascular Science University of Edinburgh Edinburgh UK; ^3^ Division of Molecular and Clinical Medicine University of Dundee Dundee UK

**Keywords:** cardiovascular function, coronary flow reserve, hypoglycaemia, myocardial ischaemia, type 1 diabetes

## Abstract

**Aims:**

This study examined the effect of experimentally‐induced hypoglycaemia on measures of myocardial blood flow and myocardial injury in adults with, and without, type 1 diabetes.

**Methods:**

In a prospective, randomised, open‐label, blinded, endpoint cross‐over study, 17 young adults with type 1 diabetes with no cardiovascular risk factors, and 10 healthy non‐diabetic volunteers, underwent hyperinsulinaemic‐euglycaemic (blood glucose 4.5–5.5 mmol/L) and hypoglycaemic (2.2–2.5 mmol/L) clamps. Myocardial blood flow was assessed using transthoracic echocardiography Doppler coronary flow reserve (CFR) and myocardial injury using plasma high‐sensitivity cardiac troponin I (hs‐cTnI) concentration.

**Results:**

During hypoglycaemia, coronary flow reserve trended non‐significantly lower in those with type 1 diabetes than in the non‐diabetic participants (3.54 ± 0.47 vs. 3.89 ± 0.89). A generalised linear mixed‐model analysis examined diabetes status and euglycaemia or hypoglycaemia as factors affecting CFR. No statistically significant difference in CFR was observed for diabetes status (*p* = .23) or between euglycaemia and hypoglycaemia (*p* = .31). No changes in hs‐cTnI occurred during hypoglycaemia or in the recovery period (*p* = .86).

**Conclusions:**

A small change in CFR was not statistically significant in this study, implying hypoglycaemia may require more than coronary vasomotor dysfunction to cause harm. Further larger studies are required to investigate this putative problem.

## Graphical abstract

Hypoglycaemia may require more than coronary vasomotor dysfunction to mediate harm

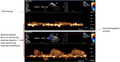

## 1 INTRODUCTION

Diabetes is now a global pandemic^1^ in which the principal cause of death is cardiovascular disease.^2^ While strict control improves some outcomes, clinical trials in people with type 2 diabetes who have cardiovascular disease have shown that intensive glucose‐lowering therapy is associated with higher mortality.^3^, ^4^ It has been suggested that exposure to hypoglycaemia may be contributing to this greater morbidity and mortality.^5^, ^6^

Hypoglycaemia is a common occurrence in people with insulin‐treated diabetes. It causes profound autonomic stimulation leading to activation of the sympatho‐adrenal system with profuse catecholamine release. This has pronounced systemic and regional haemodynamic effects,^7^ including large increments in stroke volume and cardiac output,^8^ caused by increased left ventricular ejection fraction and myocardial contractility.^9^ This augmented cardiac workload may provoke myocardial ischaemia or cardiac failure in people who have established cardiac disease.

Mechanisms that could promote adverse effects of hypoglycaemia on cardiovascular function have been proposed.^10^ Endothelial dysfunction is a key pathophysiological pathway underlying macrovascular complications in type 1 and type 2 diabetes. A detrimental effect of hypoglycaemia on peripheral endothelial function has been reported.^11^ However, several differences exist between peripheral and coronary arterial endothelium, such as the presence of shunt vessels and the microvascular architecture.^12^ Direct measurement of coronary vasomotor function may therefore provide a more valid measure of any cardiovascular impairment resulting from limited vascular responsiveness. Measurement of coronary microvascular dysfunction by calculation of coronary flow reserve (CFR) is the preferred investigative technique.^13^ Coronary flow reserve measures the capacity of the coronary circulation to increase flow during maximal resistance vessel vasodilatation. Maximal hyperaemia is achieved by intravenous infusion of adenosine^14^ and coronary flow velocity can be measured non‐invasively by transthoracic Doppler echocardiography.^15^, ^16^ CFR is reduced in people with type 1 diabetes during euglycaemia^17^ and in individuals with type 1 diabetes with evidence of microvascular disease in the form of retinopathy.^18^ CFR has also been shown to correlate well with long term hard outcomes in coronary heart disease.^19^, ^20^ It is unclear how hypoglycaemia affects CFR.

Cardiac troponins are a biomarker of myocardial injury. In recent years, the introduction of a highly sensitive cardiac troponin I (hs‐cTnI) assay allows the detection of small degrees of myocardial damage. Lowering the diagnostic threshold for myocardial injury has improved outcomes for people with type 1 myocardial infarction.^21^ Hs‐cTNI also provides prognostic information regarding long term outcomes in cohorts of people with underlying coronary heart disease.^22^

People with type 2 diabetes often have multiple cardiovascular risk factors that can confound attempts to investigate hypoglycaemia as a causative mechanism for cardiovascular morbidity and mortality. Younger people with type 1 diabetes of relatively short duration are less likely to have acquired these additional risk factors. To examine whether hypoglycaemia might adversely affect myocardial blood flow and cause myocardial injury, coronary flow reserve and cardiac troponins were therefore measured during experimentally‐induced hypoglycaemia in people with type 1 diabetes who had no confounding factors.

## 2 MATERIALS AND METHODS

### 2.1 Study participants

Young healthy male adults with type 1 diabetes with no microvascular complications or cardiovascular risk factors were recruited from diabetes out‐patient clinics in Lothian, Scotland. Men without diabetes, matched for age, were recruited by poster advertisements and from a database of volunteers. Only male subjects were studied to avoid the confounding effect of the variability of coronary flow reserve that occurs during the menstrual cycle.^23^ In addition, because the counterregulatory hormonal responses to hypoglycaemia differ between men and women,^24^ the study was confined to men to avoid a potential effect of gender on the magnitude of the sympatho‐adrenal stimulus during hypoglycaemia.

Exclusion criteria included co‐existent systemic disease, malignancy, chronic alcoholism, psychiatric disorder, any history of cardiac conduction abnormality, impaired awareness of hypoglycaemia (as assessed by the method of Gold et al.^25^), past history of severe hypoglycaemia, and any evidence of overt microvascular complications including retinopathy and neuropathy or the presence of microalbuminuria. The participants did not have any overt evidence of autonomic neuropathy. The use of any medications apart from insulin therapy, including beta‐blockers and angiotensin‐converting enzyme inhibitors, was an exclusion criterion.

A total of 17 male adults with type 1 diabetes and 10 age‐matched individuals without diabetes were studied (Table 1). Participants with type 1 diabetes had reasonable glycaemic control, (average glycated haemoglobin (HbA1c): 8.0 ± 1.1%; 64 + 11 mmol/mol), with a median duration of diabetes of 15 years (range 2–35 years), which is consistent with average quality of glycaemic control recorded in the adult population with type 1 diabetes in Scotland. The two groups of participants did not differ in age or body‐mass index (Table 1). The participants performed more frequent glucose testing 24 h before each of the glucose clamp studies to ensure that they had not been exposed to antecedent hypoglycaemia before the study.

**TABLE 1:** Clinical characteristics of participants with and without type 1 diabetes

	Participants with type 1 diabetes *n* = 17	Participants without diabetes *n* = 10	
Age (years) (median, range)	30 (20–35)	24.5 (21–33)	*p* = .13
Sex (% M)	100	100	
Body Mass Index (kg/m^2^) (mean ±SD)	25.9 ± 2.1	24.2 ± 2.7	*p* = .11
HbA1c (%;mmol/mol) (mean ± SD)	8 ± 1.1 (64 ± 11)	n/a	
Duration of diabetes (years) (median, range)	15 (2–35)	n/a	

The study was conducted with the informed written consent of all subjects, the approval of the Lothian Medical Research Ethics committee, and in accordance with the Declaration of Helsinki.

### 2.2 Study design

Participants attended two study visits, performed on separate days at least 2 weeks apart to avoid any potential carry‐over effects. Two experimental conditions, hypoglycaemia (blood glucose 2.5 mmol/L; 99 mg/dL) and euglycaemia (4.5 mmol/L; 81 mg/dL), were studied in a prospective, randomised, open‐label, blinded endpoint, (PROBE) cross‐over study. Both the group with type 1 diabetes and matched individuals underwent the experimental conditions, and then crossed over. The order of the experimental method was randomised using alternate randomisation by the operator of the glucose clamp, while the endpoint was blinded to the echocardiographer (Supplemental Figure S1). This study design has been employed previously by our group,^26^ with modifications for the cardiovascular investigations.

Participants attended in a fasting state, having abstained from the consumption of caffeine‐containing food and beverages for 24 h. Venous cannulae were inserted for intravenous infusion of dextrose and insulin, and blood sampling. A modified version of the hyperinsulinaemic glucose clamp was employed.^27^ To arterialise blood samples, the non‐dominant arm was wrapped in a heated blanket with a retrograde intravenous cannula inserted into the forearm. An additional cannula was inserted into the non‐dominant antecubital fossa to infuse insulin (human Actrapid; Novo Nordisk, Crawley, U.K.) and 20% dextrose. Insulin was infused at a constant rate of 1.5 mU/kg/min with a Gemini PCI pump (Alaris Medical Systems). Blood samples were taken at 5‐min intervals and analysed by a glucose oxidase method (2300 STAT; YSI, Yellow Springs, OH). The dextrose infusion rate was adjusted to maintain the appropriate arterialised blood glucose concentration. During a run‐in period, arterialised blood glucose was maintained at 4.5 mmol/L for 20 min. Blood glucose was then either maintained at 4.5 mmol/L throughout (the euglycaemia condition), or lowered over 20 min to 2.5 mmol/L (the hypoglycaemia condition), and maintained at this level for 30 min before restoration of euglycaemia. During the glucose clamp, the participants underwent an ultrasound examination by a trained ultrasound operator, using a well‐described technique.^16^, ^23^ The timepoints were labelled as *baseline*, *experimental* (either euglycaemia or hypoglycaemia‐blinded to the sonographer), and *recovery* (Supplemental Figure S1). Continuous electrocardiographic monitoring and regular blood pressure monitoring were performed throughout the study.

### 2.3 Coronary flow velocity measurements

During each study condition, the left anterior descending coronary artery was visualized by trans‐thoracic echocardiography. Transthoracic Doppler echocardiography was used for a non‐invasive estimation of coronary flow velocity (CFV), and maximal coronary vasodilatation was induced with an adenosine infusion to allow calculation of CFR. Imaging of the left anterior descending (LAD) artery and measurement of coronary blood flow velocity was performed using a 7.0 MHz transducer (Acuson Sequoia 512, Siemens Medical Solutions, Berkshire, UK). Baseline spectral Doppler signals were recorded initially in the distal portion of the LAD coronary artery over five cardiac cycles at end‐expiration. To measure coronary flow reserve (CFR), intravenous adenosine was administered (0.14 mg/kg/min; Adenocor, Sanofi) for up to 4 min to record spectral Doppler signals during hyperaemic conditions.^14^ Coronary velocities were measured at baseline and peak hyperaemic conditions from the Doppler signal recordings. Measurements were averaged over three cardiac cycles. CFR was defined as the ratio of hyperaemic to basal velocities, using maximum velocity (Vmax) parameters (Supplemental Figure S2). Blood pressure was recorded at baseline, during adenosine infusion and at recovery. CFR was calculated at baseline, during the experimental phase (0–20 min) and in the recovery phase.

### 2.4 High‐sensitivity cardiac troponin I concentration

Blood samples were taken prior to the assessment of CFR, during the experimental hyperinsulinaemic clamp, and during the recovery period (Supplemental Figure S1). High‐sensitivity cardiac troponin I concentrations were determined using the ARCHITECT *STAT* high‐sensitive troponin I assay (Abbott Laboratories). This is the first clinically approved high‐sensitivity troponin I assay, which has excellent precision at very low concentrations. The limit of detection is 1.2 ng/L and precision profiling in our laboratory has demonstrated an inter‐assay coefficient of variation (CV) of <10% at 5 ng/L. The upper reference limit or 99th centile is 16 ng/L for women and 34 ng/L for men.^28^, ^29^, ^30^

### 2.5 Statistical methods

A power calculation was performed using results from a previous study using a similar technique,^31^ a sample size of 12 allows an 80% chance of detecting a 0.57 difference in CFR, which is considered clinically relevant. The effects of hypoglycaemia on coronary flow reserve were assessed statistically by generalised linear mixed‐effects modelling, with the experimental condition (hypoglycaemia and euglycaemia) and diabetes status as variables affecting CFR. We have specified per subject intercepts in our mixed‐effects model by fitting individual subjects as a random effect to account for variation in baseline values across our study population. We also added an interaction term for diabetes status and experimental condition (hypoglycaemia or euglycaemia) in our mixed‐effects model.

Statistical significance was taken as a two‐sided *p *< .05. Unless specifically stated, results are mean ± standard deviation. The hsTnI data were log‐transformed due to the skewed distribution. Heart rate, blood pressure, glucose and troponin data were analysed using paired *t*‐tests. Analysis of the results was performed using R stats (R Foundation for Statistical Computing, Vienna Austria, version 3.6.1).

## 3 RESULTS

Study participants were all healthy young men with normal a body‐mass index (BMI). Baseline characteristics of both groups are provided in Table 1. During the hypoglycaemia session (Table 2), the mean glucose nadir was 2.34 ± 0.2 mmol/L; 42.12 + 3.8 mg/dL (*p* < .001 compared to baseline), which is sufficient to stimulate a brisk sympatho‐adrenal counterregulatory response. Heart rate, systolic and mean blood pressure did not change during euglycaemia in either group. During hypoglycaemia, a trend was apparent towards an increase in heart rate and systolic blood pressure (BP), and a decrease in diastolic BP in both groups (Table 2). In the type 1 diabetes group, the increments in heart rate and systolic blood pressure did not reach statistical significance. In the non‐diabetic group, the heart rate increased from 71 ± 9 beats per minute (bpm) to 78 ± 8 bpm (*p* = .02) while the systolic BP increased from 116 ± 11 to 124 ± 12 mmHg (*p* = .001).

**TABLE 2:** Plasma glucose, heart rate, systolic, and diastolic blood pressure values during hypoglycaemia and euglycaemia in the groups of participants, with and without type 1 diabetes

	Euglycaemic Clamp				Hypoglycaemic clamp		
		Baseline	Experimental	*p* value	Baseline	Experimental	*p* value
	Glucose (mmol/L)	4.95 ± 0.65	4.70 ± 0.42	.13	4.89 ± 0.62	2.34 ± 0.21	<.0001
People with type 1 diabetes	Heart rate (bpm)	73 ± 14	73 ± 8	.68	66 ± 12	70 ± 9	.2
	Systolic BP (mmHg)	127 ± 14	131 ± 13	.58	127 ± 14	130 ± 9	.43
	Diastolic BP (mmHg)	74 ± 6	71 ± 6	.84	72 ± 6	70 ± 6	.03
People without type 1 diabetes	Heart rate (bpm)	74 ± 13	76 ± 10	.73	71 ± 9	78 ± 8	.02
	Systolic BP (mmHg)	124 ± 15	126 ± 13	.61	116 ± 11	124 ± 12	.001
	Diastolic BP (mmHg)	69 ± 6	69 ± 6	.52	67 ± 5	64 ± 5	.2

### 3.1 Coronary flow velocities and reserve

No differences were observed between the coronary flow velocities of the group with type 1 diabetes and the non‐diabetic group at baseline (Supplemental Figure S3). During hyperaemia after adenosine infusion, a trend for lower coronary flow velocities was observed in people with type 1 diabetes, but this did not reach statistical significance (Supplemental Figure S3).

**FIGURE 1:**
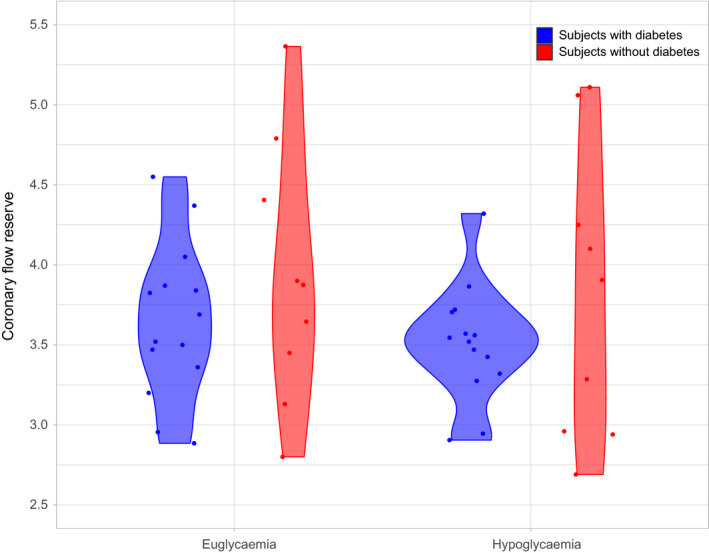
CFR (scatter and violin plot) in participants with type 1 diabetes and people without diabetes under euglycaemic and hypoglycaemic conditions. Blue represents people with diabetes, and red for people with diabetes. During euglycaemia the mean CFR for people with diabetes versus without was 3.66 ± 0.47 versus 3.92 ± 0.85, respectively. During hypoglycaemia the mean CFR was 3.54 ± 0.47 versus 3.89 ± 0.89, respectively

During euglycaemia, a trend for a lower coronary flow reserve was observed in young adults with type 1 diabetes, compared to people without diabetes (3.66 ± 0.47 vs. 3.92 ± 0.85), but this did not achieve statistical significance. During hypoglycaemia, coronary flow reserve trended non‐significantly lower in those with type 1 diabetes than in the non‐diabetic participants (3.54 ± 0.47 vs. 3.89 ± 0.89) (Figure 1). From the generalised linear mixed model analysis, no statistical significance was reached for euglycaemia or hypoglycaemia affecting CFR (*p* = .31). No statistical significance was noted for the effect of diabetes status on CFR (*p* = .23). This exploratory model did not demonstrate any significant interaction between diabetes status and experimental condition (*p* value = .938).

### 3.2 High‐sensitivity cardiac troponin I

**FIGURE 2:**
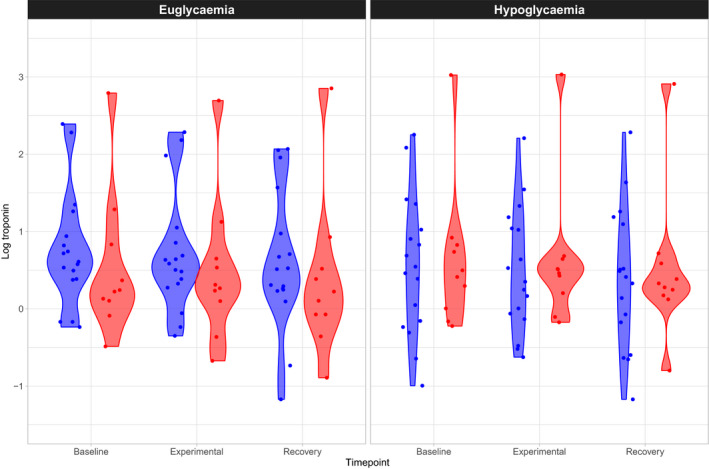
Log transformed highly sensitive troponin I values in ng/L (violin and scatter plot), during baseline, experimental condition and recovery, for participants with type 1 diabetes and people without diabetes. Participants with type 1 diabetes are in blue and those without diabetes in red

The hs‐cTnI values were in a skewed distribution and were, therefore, log‐transformed for statistical analysis. No changes were observed in plasma high‐sensitivity cardiac troponin I concentrations during euglycaemia or hypoglycaemia during the recovery phase in either group (Figure 2).

## 4 DISCUSSION

The present study used a well‐validated, non‐invasive method to examine the effect of acute hypoglycaemia on real‐time coronary arterial flow in adults with and without type 1 diabetes, with the coronary flow ratio (CFR) being measured using transthoracic Doppler echocardiography. During acute hypoglycaemia, young adult males with type 1 diabetes had a trend of lower coronary flow reserve compared with an age‐matched group of non‐diabetic males. The modest decline in CFR was well tolerated in young men with type 1 diabetes who were otherwise healthy and had no evidence either of microvascular complications or coronary heart disease; normal coronary reserve was maintained.

The direct effects of hypoglycaemia on cardiac function have proved difficult to elucidate as insulin *per se* exerts direct effects on the heart. Fisher et al.^9^ showed that administration of insulin caused an immediate increase in left ventricular ejection fraction and provoked sympathetic activation, both of which occurred before any fall in blood glucose. As blood glucose declined progressively, these responses became more pronounced, with the maximal changes coinciding with the glucose nadir.^9^ A strength of the present study was the ability to use a non‐invasive real‐time assessment of coronary flow reserve during acute hypoglycaemia. By using a hyperinsulinaemic glucose clamp it was possible to compare CFR during euglycaemia and hypoglycaemia, and between type 1 diabetes and the non‐diabetic state. The changes observed are therefore related to the low blood glucose and counterregulatory mechanisms, and not to the intravenous infusion of insulin *per se*. Furthermore, this study provides a direct assessment of coronary vasomotor function which excludes other potential confounders such as hypertension or microvascular disease.

The main limitation of the study is the small sample size. While this was in part a consequence of the robust exclusion criteria, the demanding study design also limited recruitment of potential participants, specifically because of the use of adenosine to induce maximal hyperaemia of the coronary blood vessels. The rationale for using adenosine was its short half‐life, which meant that the CFR values from one measurement to the next would not be confounded by residual effects of adenosine. The disadvantage of this approach is that adenosine is often poorly tolerated because it induces unpleasant side effects of chest tightness and facial flushing, which diminishes the willingness of volunteers to participate. In addition, the glucose clamp procedure is onerous and had to be repeated at least 2 weeks apart, which was a further limitation to recruitment.

In retrospect, the power calculation may have benefited from an *a priori* calculation of possible attrition of participants, or non‐concordance with the protocol. Additionally, the magnitude of the primary outcome (difference of 0.57 in CFR) may have been over‐ambitious, given that our participants with diabetes were fit and in good health. In view of the relatively small sample size of our study, it is possible that we did not have sufficient power to detect a significant interaction between diabetes status and experimental condition in the generalised linear mixed model analysis. Therefore, while this study models the direct effect of hypoglycaemia *per se* on coronary vasomotor function, the generalisability of the results must take into account the sample size and the exclusion of female participants.

A previous investigation by Rana and colleagues,^32^ which to our knowledge is the only other study to have explored the effect of acute hypoglycaemia on the myocardial circulation, used sequential hyperinsulinaemic glucose clamps and dipyridamole‐induced stress echocardiography to measure myocardial blood flow reserve during euglycaemia and acute hypoglycaemia in adults of both sexes, 28 with, and 19 without type 1 diabetes.^32^ The age range was wider than in the present study and included people with microvascular disease. Hypoglycaemia induced a significant fall in myocardial blood flow reserve in both groups, with lower values being observed in the group with type 1 diabetes at all times of measurement. A statistically significant association was observed with the presence of microvascular complications. In contrast to the present study design, no time interval was allowed between the initial euglycaemia and the subsequent induction of hypoglycaemia, so that myocardial blood flow reserve rose during the period of protracted euglycaemia, which may have influenced the effect on the subsequent hypoglycaemia.^32^ In addition, the order of the euglycaemic and hypoglycaemic clamps was not randomised, which may have introduced observer bias and an order effect. Furthermore, the long half‐life of dipyridamole might have influenced the results. The present study may therefore have provided a more direct model of coronary flow reserve with fewer confounding factors such as the presence of microvascular disease and a possible residual effect of dipyridamole.

In the present study, no significant change in CFR was observed during hypoglycaemia. While a non‐significant trend towards a lower CFR was observed in the participants with type 1 diabetes during euglycaemia, a trend towards a decline in CFR was also observed during hypoglycaemia, consistent with previous observations.^17^ The lowest CFR values during hypoglycaemia were observed in the participants with type 1 diabetes. The increments in heart rate and systolic blood pressure in the group with type 1 diabetes did not achieve statistical significance (Table 2), which was unexpected with this degree of hypoglycaemia. The small sample size may not have allowed sufficient sensitivity to detect small variations in pulse and blood pressure. An alternative interpretation is that some participants with type 1 diabetes may have had some degree of autonomic dysfunction or a diminished catecholamine response to hypoglycaemia, which contributed to the modest haemodynamic changes and the lower CFR values observed in the group with type 1 diabetes. As a formal assessment of autonomic function in the participants was not made nor were plasma catecholamines measured, this possibility cannot be excluded.

A Danish study using non‐invasive cardiac magnetic resonance imaging has reported that myocardial blood flow reserve was higher at rest and lower during vasodilatory stress in people with type 2 diabetes compared with non‐diabetic controls.^33^ Impaired myocardial blood flow reserve was associated with microvascular complications (albuminuria and retinopathy) of diabetes. The present study explicitly excluded people with overt microvascular disease; it is possible that the development of cardiac microangiopathy may underlie an abnormal response to hypoglycaemia in type 1 diabetes.^33^

While no change in the highly sensitive troponin values was observed during acute hypoglycaemia, this was measured in close temporal proximity to the blood glucose nadir. It is possible that measurement in the immediate ‘recovery’ stage was made too early to detect a rise in plasma troponin and exclude evidence of myocardial insult.

The results of the present study imply that any putative cardiac harm of hypoglycaemia is unlikely to be mediated solely through coronary vasomotor dysfunction. Other potentially harmful factors associated with hypoglycaemia may be required, such as the promotion of pro‐thrombotic mechanisms,^34^, ^35^ endothelial abnormalities,^11^ or altered cardiac electrical conduction.^36^, ^37^ ^,^ It should also be noted that this study specifically studied acute hypoglycaemia, and the cumulative effects of recurrent hypoglycaemia have not been examined.

Recent randomised controlled trials (RCTs) that did not target strict glycaemic control but used drugs with a low risk of hypoglycaemia have shown beneficial cardiovascular outcomes.^38^, ^39^ This is in direct contrast to previous RCTs, in which very strict glycaemic control was pursued and the incidence of severe hypoglycaemia was high.^10^ These findings suggest that avoidance of hypoglycaemia is important to achieve cardiovascular benefit.

## 5 CONCLUSION

Although in the present study hypoglycaemia had no effect on markers of ischaemia, a small reduction in CFR was apparent during hypoglycaemia, with the lowest numerical value occurring in young adults with type 1 diabetes during hypoglycaemia. Further larger studies that include female participants are required to confirm or refute this observation. If the present observation of a lower CFR is confirmed, this would raise concern that hypoglycaemia may promote myocardial ischaemia in older people with diabetes who have established coronary heart disease. While coronary blood flow should be examined during hypoglycaemia in people with type 2 diabetes who have a greater risk of cardiovascular disease, this was not undertaken because of the potential cardiac risk to such participants. The present study should be repeated in a larger number of people with type 1 diabetes, using a more tolerable form of investigation and a real time biomarker of cardiac injury. As population studies have shown an association between severe hypoglycaemia and cardiovascular risk^5^, ^40^ more mechanistic studies are required to elucidate potential reasons for this association.

## CONFLICT OF INTEREST

The authors disclose no conflicts of interest in this study.

## AUTHOR CONTRIBUTIONS

RMN, ACW, NNL and NLM contributed to the acquisition, analysis and interpretation of the data and to the preparation and critical revision of the manuscript. KKL contributed to the analysis of the data and preparation of the manuscript. DEN, CCL, AJG and BMF contributed to the study concept and design and to the preparation and critical revision of the manuscript. RMN is the guarantor of this work, had full access to the data, and takes responsibility for the integrity of the data and the accuracy of the data analysis.

## Supporting information

Fig S1

Fig S2

Fig S3

## Data Availability

The datasets used and/or analysed during the current study are available from the corresponding author on reasonable request.
