# Supplementary figures and images for: Effect of hypoglycaemia on measures of myocardial blood flow and myocardial injury in adults with and without type 1 diabetes: A prospective, randomised, open‐label, blinded endpoint, cross‐over study
Source: Endocrinol Diabetes Metab. 2021 May 7;4(3):e00258. doi: 10.1002/edm2.258 (PMC8279606; doi:10.1002/edm2.258)

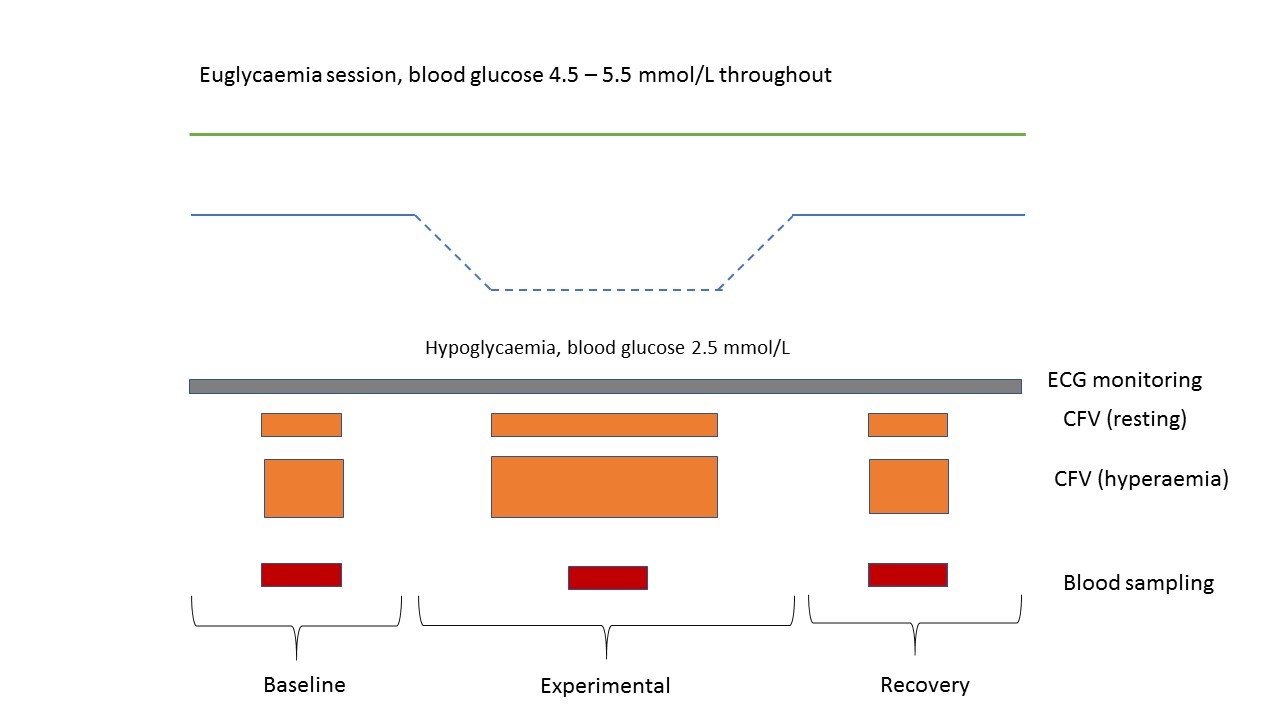

Supplement: Supplementary file 1 — Fig S1 [file EDM2-4-e00258-s001.jpg]

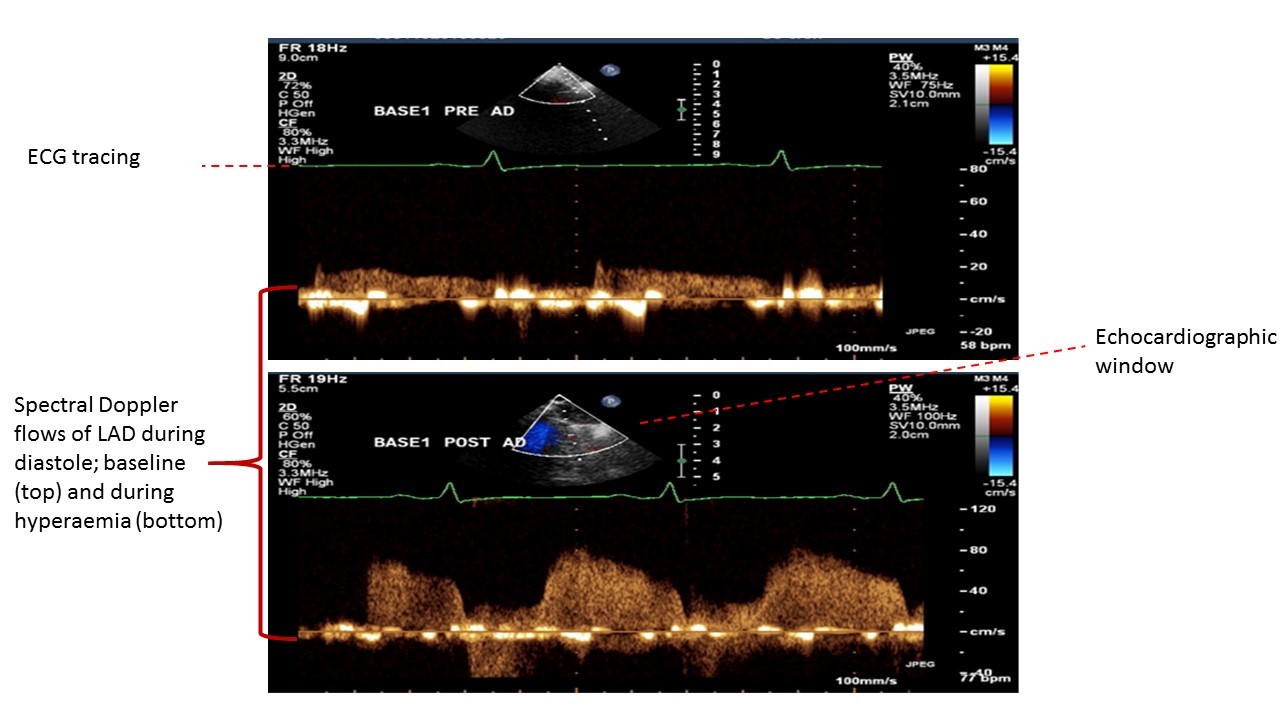

Supplement: Supplementary file 2 — Fig S2 [file EDM2-4-e00258-s002.jpg]

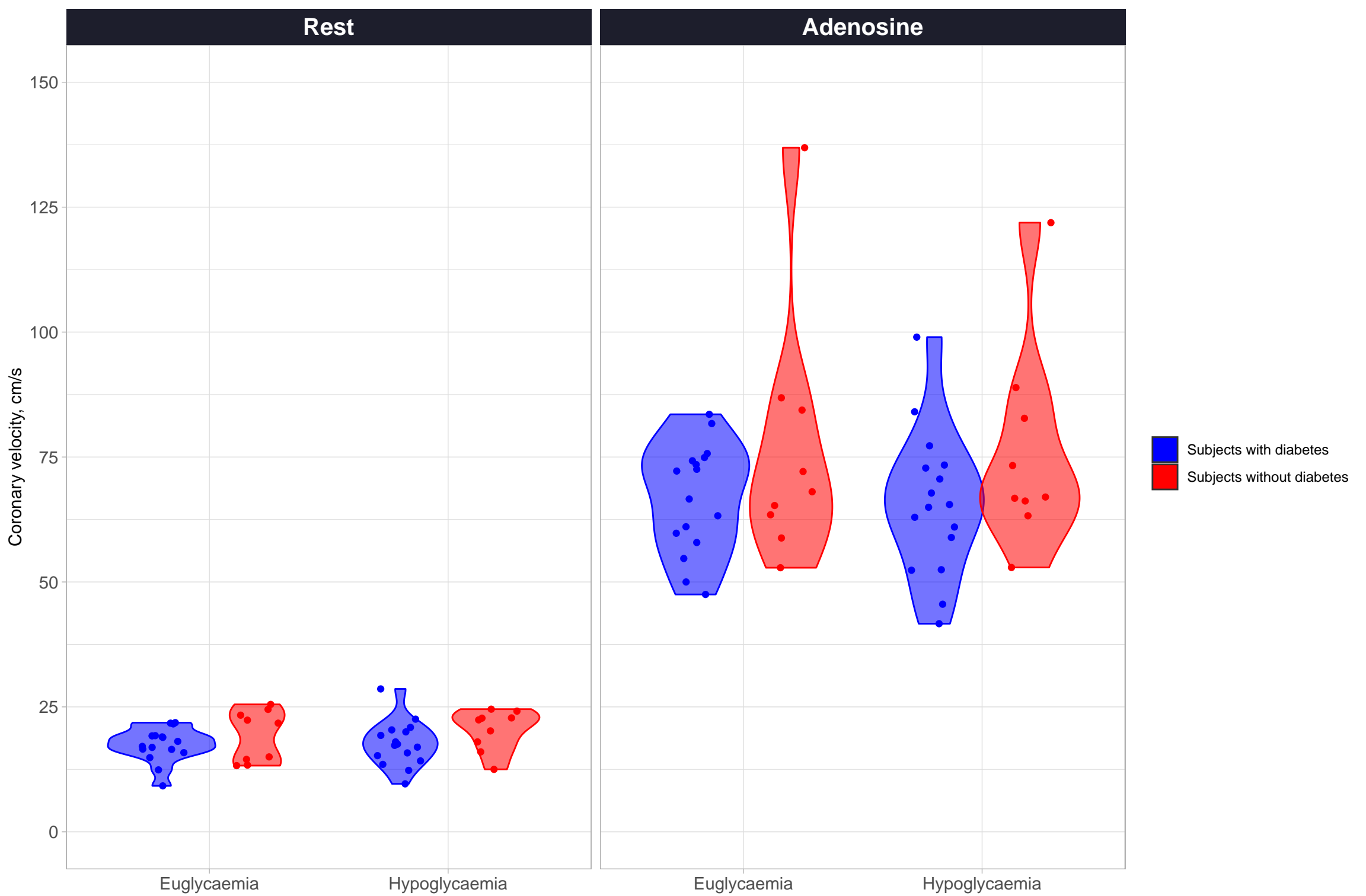

Supplement: Supplementary file 3 — Fig S3 [file EDM2-4-e00258-s003.pdf]
